# Magnetic Lu_2_Cu_2_O_5_-based ceramic nanostructured materials fabricated by a simple and green approach for an effective photocatalytic degradation of organic contamination

**DOI:** 10.1039/d1ra06101a

**Published:** 2021-12-16

**Authors:** Seyed Milad Tabatabaeinejad, Sahar Zinatloo-Ajabshir, Omid Amiri, Masoud Salavati-Niasari

**Affiliations:** Institute of Nano Science and Nano Technology, University of Kashan P. O. Box. 87317-51167 Kashan Iran salavati@kashanu.ac.ir +98 31 55913201 +98 31 55912383; Department of Chemical Engineering, University of Bonab P.O. Box. 5551761167 Bonab Iran s.zinatloo@ubonab.ac.ir; Department of Chemistry, College of Science, University of Raparin Rania Kurdistan Region Iraq

## Abstract

Designing and fabricating an efficient photocatalytic compound with an appropriate band gap to eliminate toxic contaminants is necessary to remediate the environment. This article presents the development of a new type of nanostructure, Lu_2_Cu_2_O_5_–Lu_2_O_3_ nanocomposites to photo-catalytically degrade different kinds of toxic pollutants under sunlight. The oxide nanocomposites were fabricated *via* a quick and eco-friendly approach. In order to fabricate oxide nanostructures with appropriate features in terms of morphology and particle size, the effects of the kind of green reactant and its quantity were examined. Amylum was an appropriate and green reactant for the efficient synthesis of Lu_2_Cu_2_O_5_–Lu_2_O_3_ nanobundles with the most organized morphology. The features of Lu_2_Cu_2_O_5_-based nanostructures were carefully investigated utilizing multiple characterization methods. Then, the catalytic role of the fabricated nanobundles was evaluated for the removal of various kinds of toxic contaminants. The effects of the quantity of photocatalytic nanostructure, the concentration of the contaminant compound, and the type of light source in the catalytic degradation process were screened. The findings of this research demonstrated that utilizing 0.05 g of Lu_2_Cu_2_O_5_–Lu_2_O_3_ nanobundles, 98.5% of the contaminant with a concentration of 10 ppm can be degraded in 2 h under ultraviolet light irradiation. The experimental results also certified that, during the photocatalytic pathway, superoxide radicals play a meaningful role in the elimination of toxic pollutants. To our knowledge, this is the first report of the fabrication of Lu_2_Cu_2_O_5_–Lu_2_O_3_ nanocomposite through a facile and eco-friendly approach and its photocatalytic efficiency.

## Abstract 2

The synthesis of Lu_2_Cu_2_O_5_–Lu_2_O_3_ nanocomposites *via* a simple auto-combustion approach and their excellent performance in the removal of organic pollutants under sunlight is presented for the first time.

## Graphical abstract

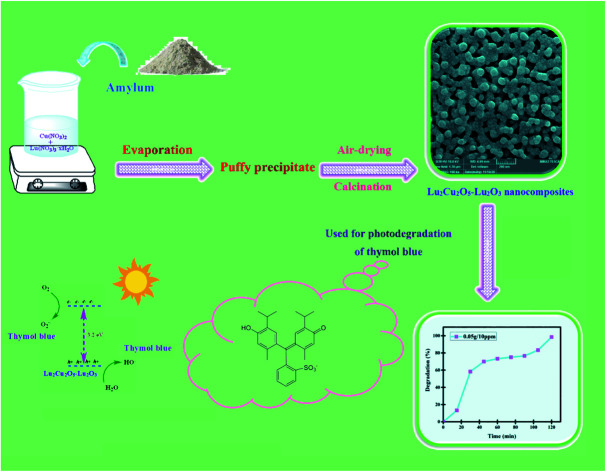

## 1. Introduction

Wastewater effluents have been reported to originate from domestic use as well as from various industries and are often comprised of significant quantities of chemical and toxic compounds, including dyes, which can trigger serious environmental problems.^1–3^ The presence of organic and toxic contaminants in the environment can be a great danger to both human life and wildlife.^4–6^ Various approaches have been utilized to remediate toxic contaminants.^7–9^ In the process of photocatalysis, a completely environmentally friendly method utilizing free sunlight in the presence of photocatalytic compounds, poisonous contaminants can be degraded or oxidized.^10–12^ Features such as non-toxicity, superior efficiency, cost-effectiveness, and the creation of safe secondary compounds have made photocatalysis an appealing method for many scientists for the remediation of environmental contaminants.^13–15^ So far, a wide variety of photocatalysts have been utilized to remove these toxic residues.^16–18^ However, there is still the challenge of designing and fabricating a photocatalytic compound with an appropriate band gap to eliminate a wide range of toxic contaminants. Thus, there is great interest in producing an efficient photocatalytic compound that can remove a broad spectrum of toxic pollutants to remediate the environment. The photocatalysis technique has undergone ultra-rapid development during the past several decades, exhibiting unique merits including stable redox ability, excellent renewability, and a green and sustainable nature, and has been widely applied in many fields such as photo-synthesis, pollutant treatment, and other chemical or energy-related processes.^19–21^ Recently, photocatalysis has attracted much interest for using solar energy to convert chemical energy, such as in H_2_ production,^22^ degradation of various kinds of pollution,^23^ H_2_O_2_ generation, and CO_2_ reduction.

Recently, the use of green chemistry-based approaches to prepare a variety of nanoscale oxide compounds has become an important research topic.^24–26^ This environmentally friendly technology utilizes non-hazardous reactants that do not lead to the problem of environmental contamination. The qualities of energy-saving, reproducibility and simplicity are also prominent features of this efficient technology.^24^

Binary lanthanide cuprates (Ln_2_Cu_2_O_5_) are known for their distinctive features that make them viable for a variety of uses, such as superconductors, magnetocaloric compounds, and sensors.^27–29^ Ln_2_Cu_2_O_5_ has been reported to crystallize as an orthorhombic phase with space group *Pna*2.^30–32^ The reported fabrication approaches of binary lanthanide cuprates have been limited to co-precipitation and solid-state reactions.^29^ The use of these approaches cannot lead to the fabrication of oxide compounds with control of their particle morphology and dimensions. Thus, it is indispensable to design easy, reproducible, energy-saving, and fast approaches for the production of Ln_2_Cu_2_O_5_ with controlled features. To our knowledge, no research work has reported on the use of a simple, quick, and environmentally friendly approach to fabricate Lu_2_Cu_2_O_5_-based nanostructured materials by employing green reactants. Here, employing soluble carbohydrates as green fuel, we report the fabrication, through a quick and eco-friendly approach, of a new type of nanostructure, Lu_2_Cu_2_O_5_–Lu_2_O_3_ nanocomposites, and their use for the photocatalytic elimination of toxic contaminants in water.

## 2. Experimental

### 2.1. Materials

Analytical reagent grade fructose, lutetium(iii) nitrate, amylum, copper nitrate, and maltobiose were purchased from Merck. The reagents were employed directly without any purification. The Lu_2_Cu_2_O_5_–Lu_2_O_3_ nanocomposite was investigated utilizing a variety of techniques.

### 2.2. Physical instrument

The morphologies of the samples were obtained using a TESCAN BRNO-Mira3 LMU field emission scanning electron microscope. The samples were covered by a thin layer of gold (Au) before taking images to achieve better contrast and bypass charge accretion. An X-ray diffractogram (Philips) with X'Pert Pro sieved by copper K_a_ radiation (*λ* = 15.4 nm) was used to record the XRD patterns. X'Pert HighScore Plus software was applied to distinguish the compounds. The FT-IR spectra of the samples were obtained on a Shimadzu Varian 4300 spectrometer in the range of 400–4000 cm^−1^ in KBr pellets. Energy-dispersive spectroscopy (EDS) with a 20 kV exciting charge was utilized for the elemental analysis of the products. The UV-visible spectrum was registered utilizing a JASCO UV-Vis scanning spectroscope (V-670). The GC-2550TG (Teif Gostar Faraz Company, Iran) was used for each alchemical agent. The surface areas (BET) were characterized *via* nitrogen adsorption at −196 °C employing automated gas adsorption analysis equipment (Tristar 3000, Micromeritics). Transmission electron microscopy (TEM) was executed on a JEM-2010 UHR JEOL transmission electron microscope (UHR-TEM) for the first batch, a FEI Tecnai G2 F20 for the second batch, and a Thermo Fischer Talos F200X for the last batch of pictures.

### 2.3. Synthesis of Lu_2_Cu_2_O_5_–Lu_2_O_3_ nanocomposite

**Scheme 1:**
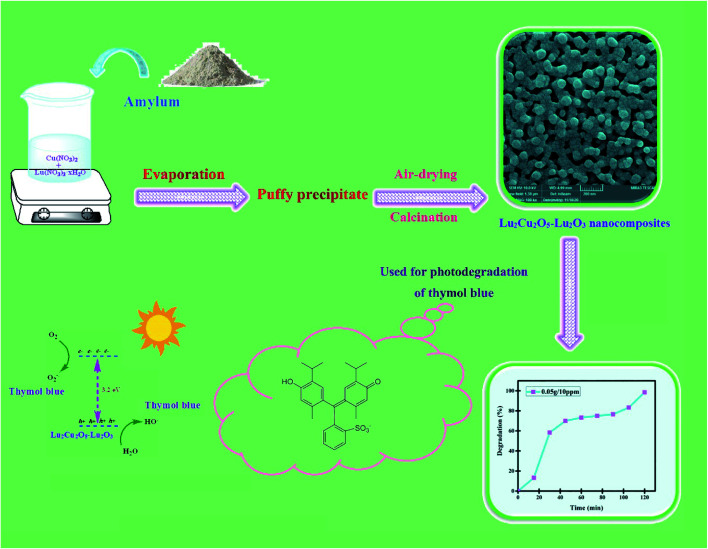
Schematic diagram illustrating the production of Lu_2_Cu_2_O_5_–Lu_2_O_3_ nanobundles and their photocatalytic degradation performance.

An eco-friendly approach was utilized to fabricate Lu_2_Cu_2_O_5_–Lu_2_O_3_ nanocomposites. First, an aqueous solution containing 5 mmol of carbohydrate (amylum) was slowly added to a solution containing 1 mmol of lutetium(iii) nitrate as well as 2 mmol of copper nitrate and the mixture was stirred for half an hour at about 50 °C. By heating the mixture to a temperature above the evaporation temperature, a puffy precipitate was created. The nanocomposite sample was prepared by calcination of the precipitate at 900 °C for 4 hours (Scheme 1). The effects of the type of green reactant and its quantity on the features of the oxide nanocomposites in terms of morphology and particle size were examined (Table 1).

**Table 1:** The production conditions for all samples

Sample no.	Green reactant type	Amount of green reactant (mmol)	Figure for FESEM images
1	Fructose	5	1a and b
2	Maltobiose	5	1c and d
3	Amylum	5	1e and f
4	Amylum	7	2a and b
5	Amylum	3	2c and d

### 2.4. Photocatalysis experiments

The catalytic role of the Lu_2_Cu_2_O_5_–Lu_2_O_3_ nanobundles was evaluated in the removal of various kinds of toxic contaminants. In each test, 0.04 g of the nanoscale photocatalyst was mixed with a solution containing 0.001 g of toxic contaminant and then stirred in the dark for half an hour to achieve equilibrium between adsorption and desorption. After that, the light source (400 W mercury lamp) was turned on.^18^ The degradation efficiency of the toxic contaminant was estimated by employing the following equation^12,18^1
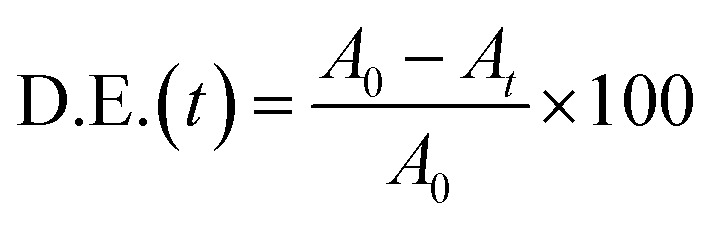
where *A*_0_ and *A*_*t*_ denote the absorption of the contaminant solution before and after irradiation. The effectiveness of the quantity of nanocatalyst, the concentration of the contaminant compound and the type of light source in the catalytic degradation process were carefully examined (Table 2).

**Table 2:** Photocatalytic degradation conditions of toxic pollutant by Lu_2_Cu_2_O_5_–Lu_2_O_3_ nanocomposites

Type of pollutant	Quantity of nanocatalyst (g)	Contaminant concentration (ppm)	Scavenger	Removal (%)
Rhodamine B	0.03	10	—	25.9
Eriochrome Black T	0.03	10	—	32.0
Thymol blue	0.03	10	—	70.0
Acid yellow	0.03	10	—	25.0
Methyl orange	0.03	10	—	8.7
Acid red	0.03	10	—	50.0
Thymol blue	0.03	15	—	96.6
Thymol blue	0.03	20	—	75.0
Thymol blue	0.05	10	—	98.5
Thymol blue	0.05	15	—	21.4
Thymol blue	0.05	20	—	33.3
Thymol blue	0.07	10	—	70.4
Thymol blue	0.07	15	—	96.6
Thymol blue	0.07	20	—	50.0
Thymol blue	0.05	10	Benzoquinone	25.2
Thymol blue	0.05	10	Benzoic acid	80.0
Thymol blue	0.05	10	EDTA	75.7

### 2.5. Scavenging investigation

Scavenging experiments were undertaken separately to detect the reactive radical species that are mainly responsible for decomposing the thymol blue. One mmol of each scavenger was employed. Benzoquinone, EDTA, and benzoic acid were employed to remove O_2_^−^˙, h^+^, and OH˙, respectively.^12^

## 3. Results and discussion

### 3.1. Microstructure and morphology

**Fig. 1:**
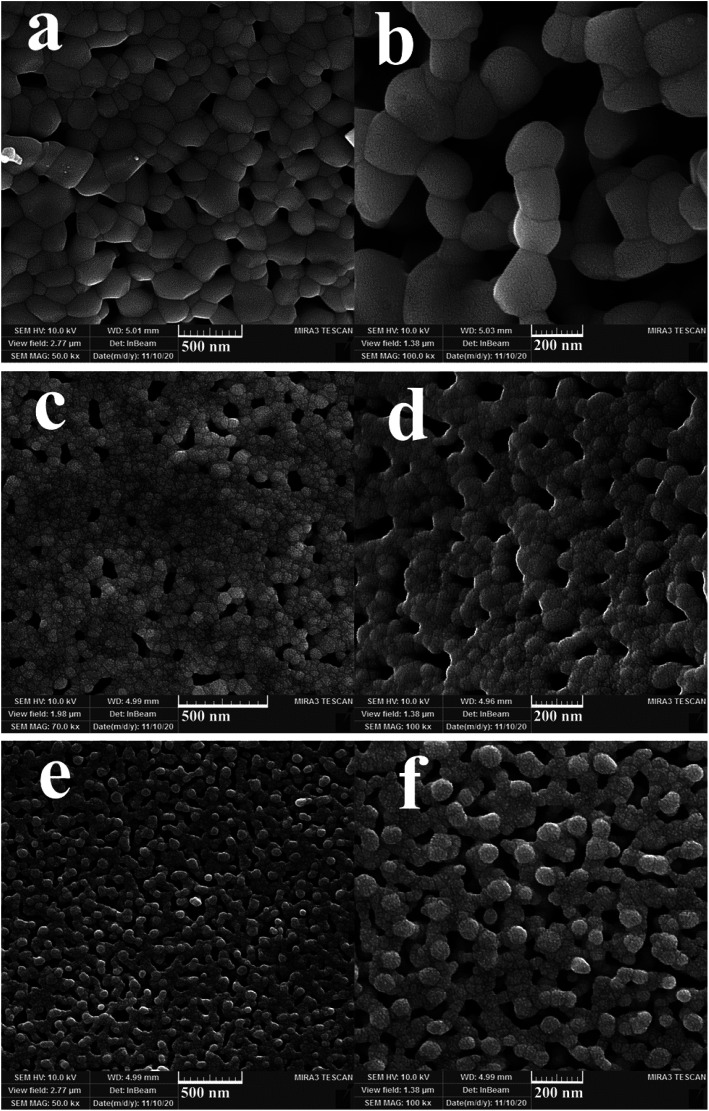
FESEM images of samples 1, 2 and 3 produced with fructose (a and b), maltobiose (c and d) and amylum (e and f).

**Fig. 2:**
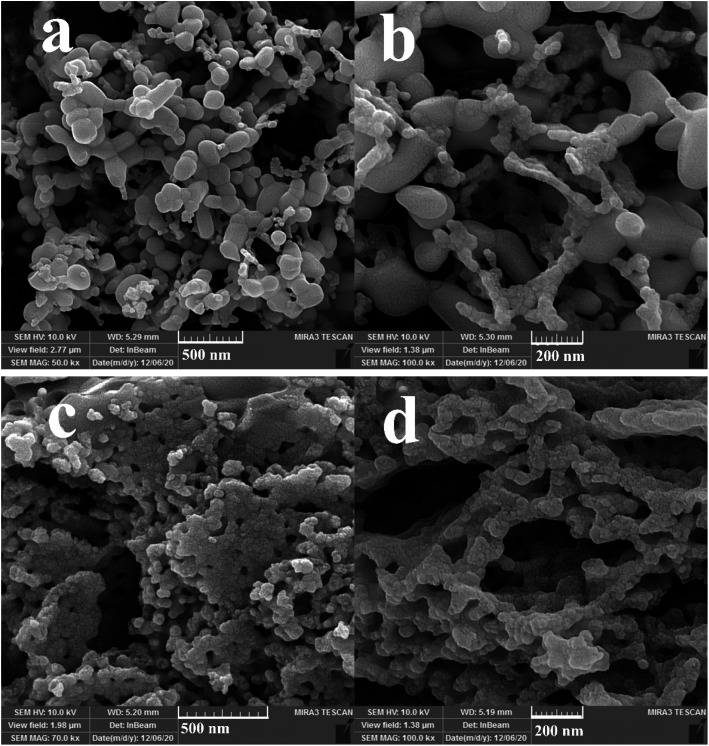
FESEM images of samples 4 and 5 produced with 7 (a and b) and 3 (c and d) mmol of amylum.

Three different types of green reagents, namely fructose, maltobiose and amylum, were employed to prepare nanocomposite samples 1, 2, and 3, respectively, to select the option which best leads to the fabrication of an oxide nanostructure with desirable features in terms of morphology and particle size (Fig. 1 and 2). As can be seen, the use of the monosaccharide carbohydrate fructose resulted in the production of irregular micro/nanostructures (Fig. 1a and b). Bundle-like nanostructures with low uniformity and high aggregation were prepared in the presence of the disaccharide carbohydrate maltobiose (Fig. 1c and d), while the use of the polysaccharide carbohydrate amylum resulted in nanobundles with the most organized morphology (Fig. 1e and f). In the process of preparing the oxide samples, all three kinds of carbohydrates played simultaneous roles as reductant and fuel and the available nitrate played the role of oxidant. All three green reactants have hydroxyl groups that can form a chelating complex with the metal ions in the reaction mixture, affecting the crystalline facets of the oxide sample and resulting in the creation of different oxide structures.^33^ It seems that amylum, which has more hydroxyl groups, is more effective in fabricating an oxide nanostructure with a more uniform morphology.^33^

**Fig. 3:**
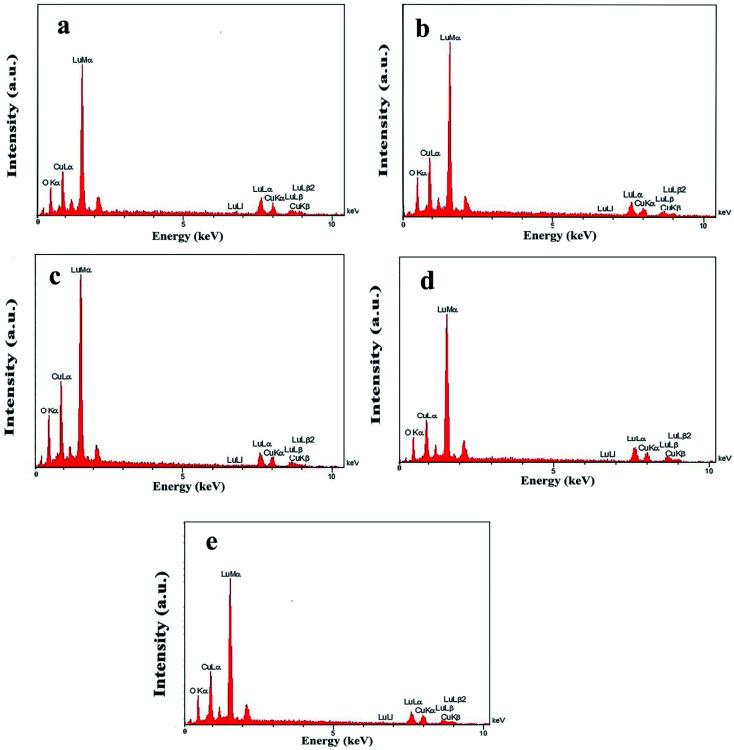
EDS patterns of samples 1 (a), 2 (b), 3 (c), 4 (d), and 5 (e).

In addition, other doses of amylum, including 7 and 3 mmol, were employed to fabricate nanocomposite samples 4 and 5 to select the most appropriate dose that could lead to a more homogeneous nanostructure. It is observed that when increasing the amylum dose to 7 mmol (Fig. 2a and b), irregular oxide microstructures are formed. Changing the amylum dose to 3 mmol (Fig. 2c and d) results in particle-like structures with high aggregation. Based on the above results, it can be concluded that the use of 5 mmol of amylum is more suitable for the creation of a uniform oxide nanocomposite in terms of morphology (Fig. 1e and f). The presence of all expected elements in the EDS study of samples prepared in this research verifies the production of oxide nanocomposites. It can be seen in Fig. 3 that elements including lutetium, oxygen, and copper are present in all prepared samples.

**Fig. 4:**
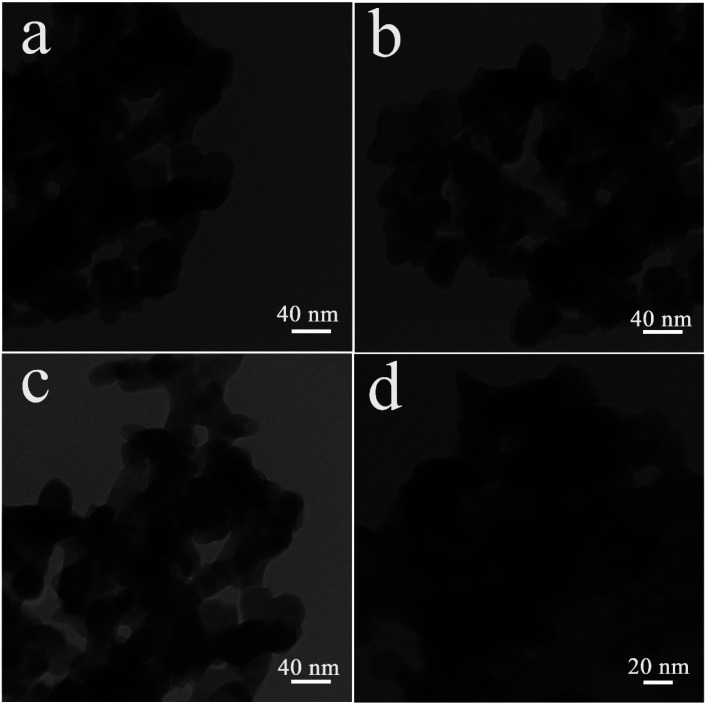
TEM images of Lu_2_Cu_2_O_5_–Lu_2_O_3_ nanobundles fabricated with 5 mmol of amylum.

The oxide sample fabricated with 5 mmol of amylum was selected for further studies. TEM images of sample 3 display its microstructure in more detail (Fig. 4). It is observed that the oxide sample is comprised of sphere-shaped particles with a size of about 20 to 40 nanometers.

### 3.2. XRD and FTIR studies

**Fig. 5:**
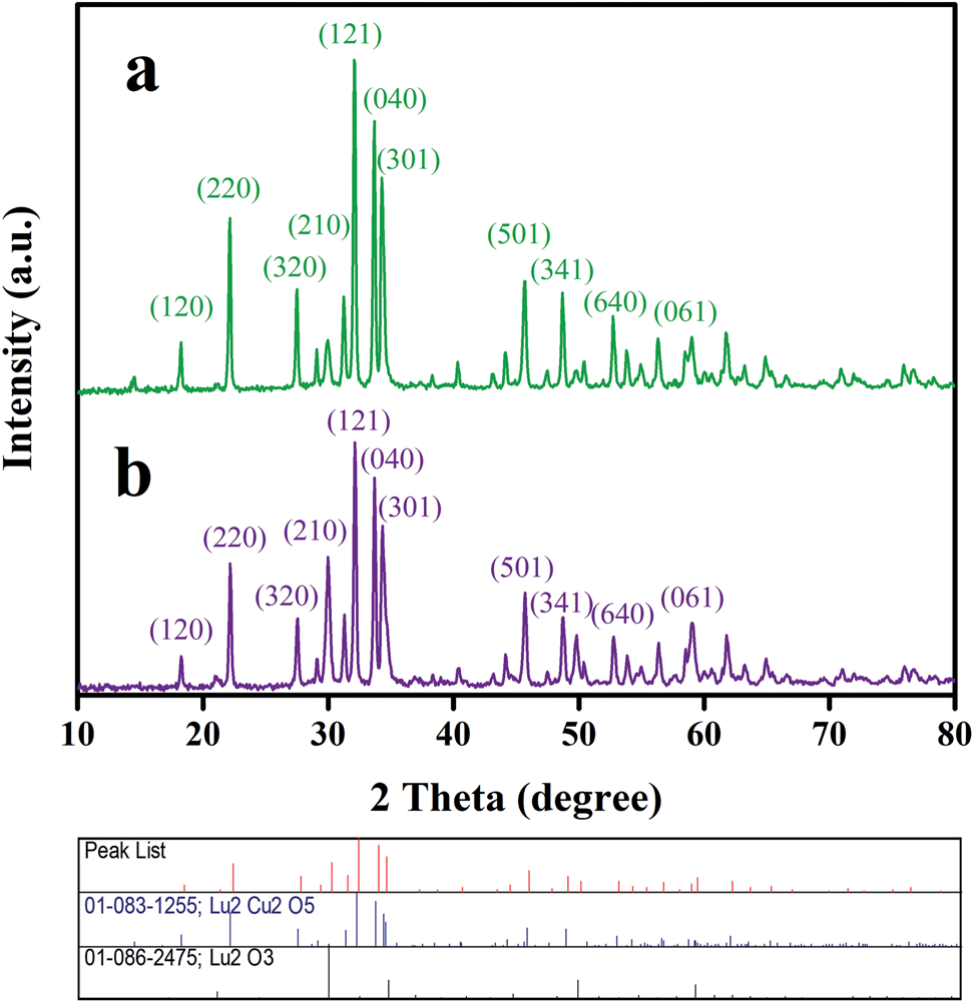
XRD patterns of samples 1 and 3 produced with fructose (a) and amylum (b), respectively.

The crystal structures of samples 1 and 3 fabricated utilizing environmentally friendly reactants, including fructose and amylum, were identified by their corresponding XRD patterns (Fig. 5). In the diffractograms of both oxide samples, the characteristic diffraction lines of Lu_2_O_3_ (JCPDS no. 01-086-2475) and Lu_2_Cu_2_O_5_ (JCPDS no. 01-083-1255) can be found, which clearly verifies the fabrication of a Lu_2_Cu_2_O_5_–Lu_2_O_3_ binary composite. No diffraction lines indicating impurities were observed. The mean crystalline sizes of both oxide samples were calculated employing the well-known Scherrer formula.^26^ It was observed that the mean crystalline sizes for the fabricated oxide samples were about 36 nm (sample 1) and 34 nm (sample 3). Thus, when the green reactant is changed from fructose to amylum, the mean size of the crystals diminishes.

**Fig. 6:**
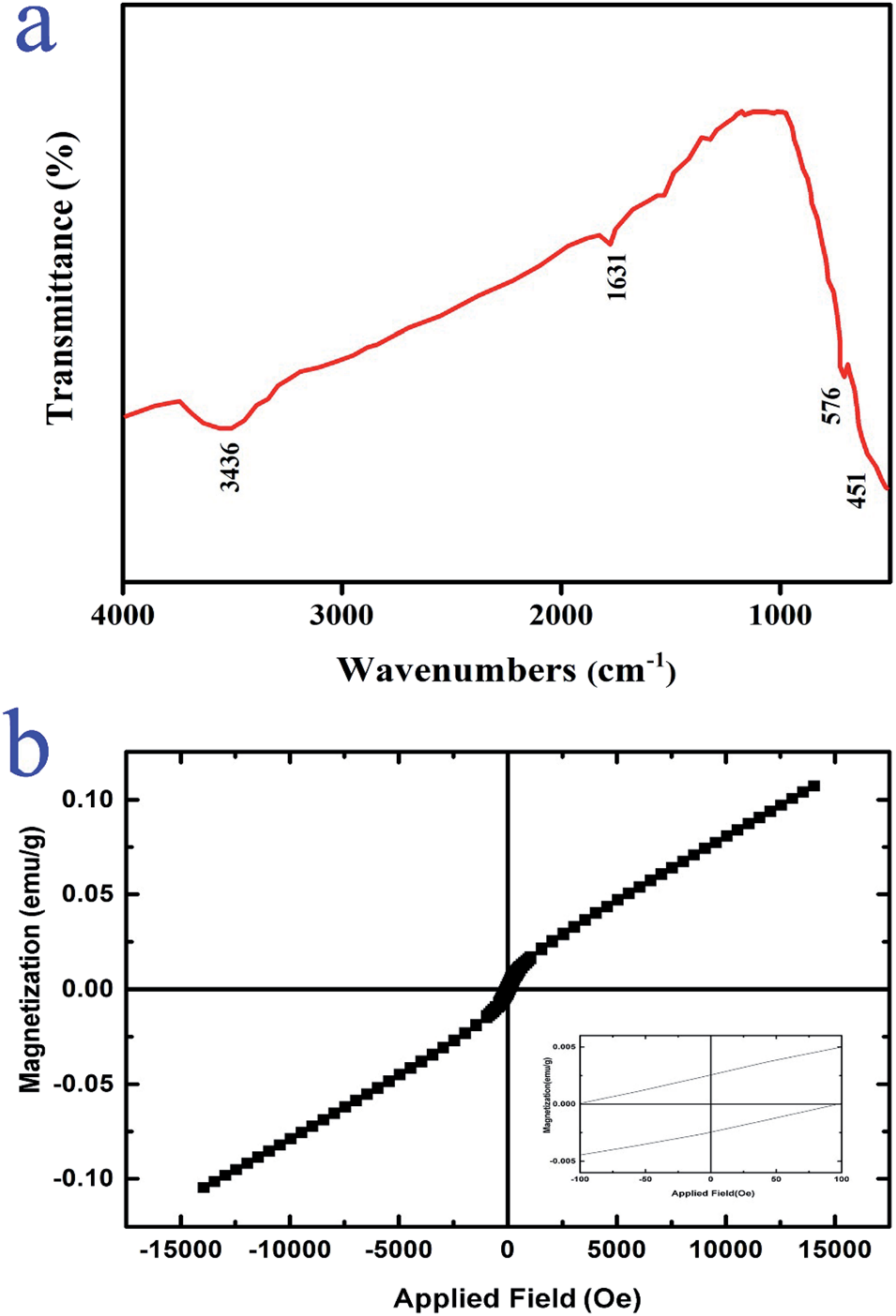
(a) FT-IR spectrum and (b) VSM results of Lu_2_Cu_2_O_5_–Lu_2_O_3_ nanobundles fabricated with 5 mmol of amylum.

Fig. 6a exhibits the FTIR spectrum of the Lu_2_Cu_2_O_5_–Lu_2_O_3_ nanobundles. The two peaks located around 576 and 451 cm^−1^ are related to the metal–oxygen bonds.^34,35^ There are two peaks near 3436 and 1631 cm^−1^ which demonstrate the presence of surface adsorbed water molecules.^18^ These results verified the successful fabrication of Lu_2_Cu_2_O_5_–Lu_2_O_3_ nanocomposites utilizing an eco-friendly approach.

### 3.3. VSM and BET studies

The magnetic features of Lu_2_Cu_2_O_5_–Lu_2_O_3_ nanobundles were examined at 300 K. It is observed that the nanocomposite sample displays dual behavior. In low fields, it has weak ferromagnetic behavior, while in high fields, it exhibits paramagnetic behavior (Fig. 6b).^36^ Having magnetic properties is an advantage for nanoscale photocatalytic compounds for environmental usage because it allows for easy recycling.^37,38^

**Fig. 7:**
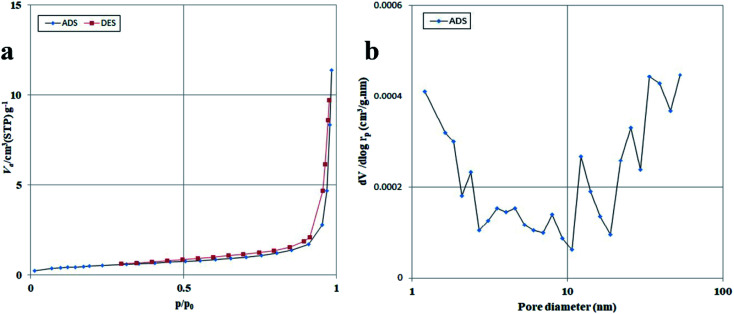
N_2_ adsorption/desorption isotherm (a) and pore size distribution curve (b) of Lu_2_Cu_2_O_5_–Lu_2_O_3_ nanobundles fabricated with 5 mmol of amylum.

The specific surface area and porosity of the photocatalytic compounds are also significant features because they can greatly affect performance.^39^ The BET–BJH profiles of the Lu_2_Cu_2_O_5_–Lu_2_O_3_ nanobundles are exhibited in Fig. 7. It is clear that the nanocomposite sample has a structure containing both meso- and macropores. Lu_2_Cu_2_O_5_–Lu_2_O_3_ nanobundles exhibit a surface area of ∼1.76 m^2^ g^−1^, with a mean pore size of ∼40 nm and a total pore volume of 0.0176 cm^3^ g^−1^. Thus, the nanocomposite sample has different pores that can be active areas for the adsorption–desorption of contaminant molecules.

### 3.4. Optical properties

**Fig. 8:**
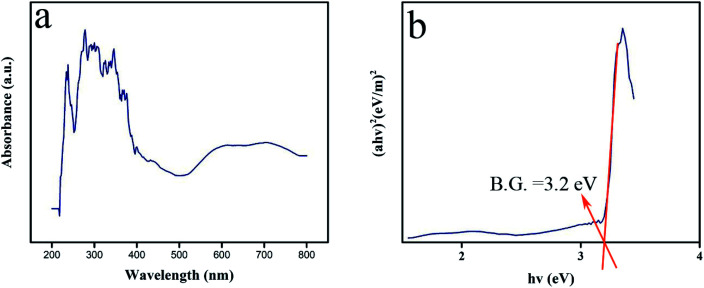
DRS spectrum (a) and plot to determine the band gap (b) of Lu_2_Cu_2_O_5_–Lu_2_O_3_ nanobundles fabricated with 5 mmol of amylum.

In photocatalytic reactions, the optical band gap of the oxide compound can play a meaningful role in the ability to harvest light. In the DRS spectral plot, Lu_2_Cu_2_O_5_–Lu_2_O_3_ nanobundles display a peak of about 276 nm (Fig. 8). The band gap can be found from the Tauc plot drawn from DRS absorbance results.^12^ The Tauc plot of Lu_2_Cu_2_O_5_–Lu_2_O_3_ nanobundles exhibits a direct band gap near 3.2 eV (Fig. 8). Due to this feature, the nanocomposite sample can be employed as a photocatalytic compound for environmental uses because it can be easily activated by light.

### 3.5. Photocatalytic degradation evaluation

**Fig. 9:**
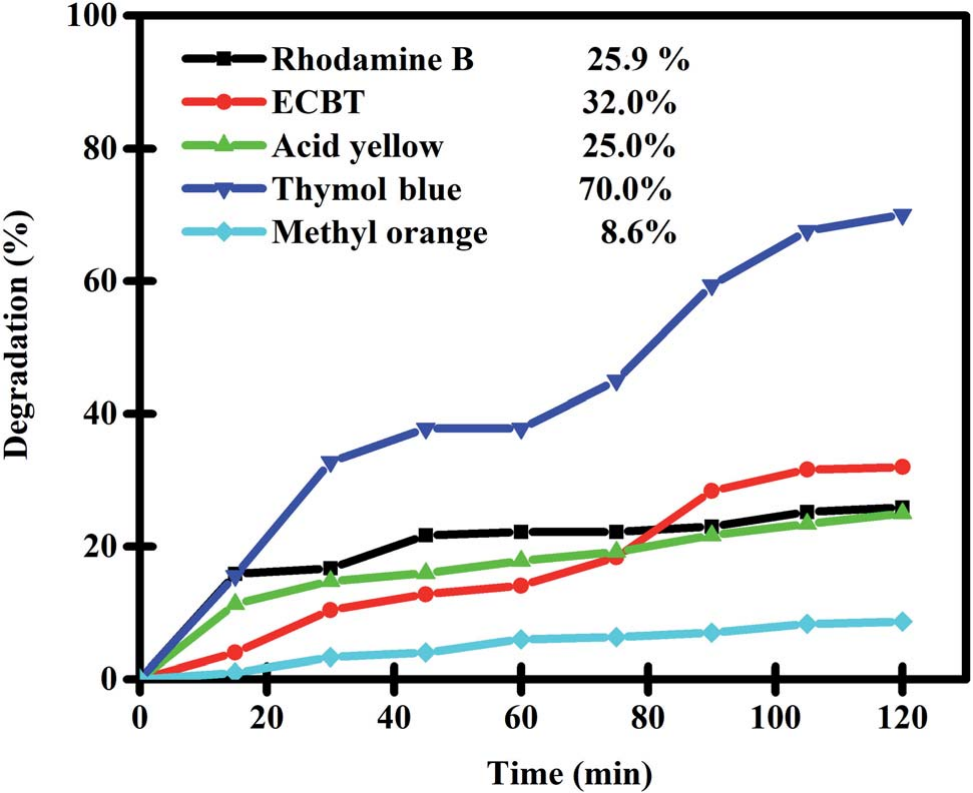
Photocatalytic degradation of various pollutants in the presence of Lu_2_Cu_2_O_5_–Lu_2_O_3_ nanobundles under UV light irradiation.

A nanocomposite sample prepared by an environmentally friendly approach (sample 3) was employed as a nanophotocatalyst to evaluate its ability to degrade toxic contaminants. The presence of organic and toxic contaminants such as dyes in the environment can be a great danger to human life and wildlife. Purifying water of these toxic contaminants is a great challenge. Hence, we selected a variety of organic dyes (including rhodamine B, Eriochrome Black T, thymol blue, acid yellow, methyl orange, and acid red) and evaluated the photocatalytic performance of Lu_2_Cu_2_O_5_–Lu_2_O_3_ nanobundles to purify water of these contaminants under ultraviolet light (Fig. 9 and Table 2). In each test, 0.03 g of the nanocomposite sample was employed for the purification of a solution with a concentration of 10 ppm of each contaminant. It is interesting to note that without light irradiation or without the presence of Lu_2_Cu_2_O_5_–Lu_2_O_3_ nanobundles, almost no dye contaminants were removed after 120 minutes, which confirms an insignificant self-destructive contribution.^18^ It was observed that the nanocomposite sample works best for the elimination of thymol blue and can remove 70% of it in 120 minutes.

**Fig. 10:**
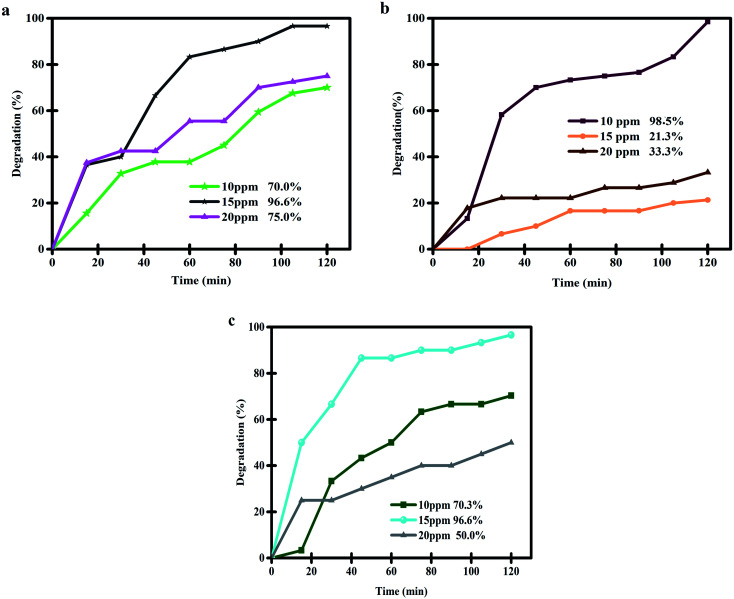
Effects of the quantity of photocatalytic nanostructure (0.03, 0.05, and 0.07 g) and the concentration of contaminant compound in the photocatalytic degradation of thymol blue under UV light irradiation.

**Fig. 11:**
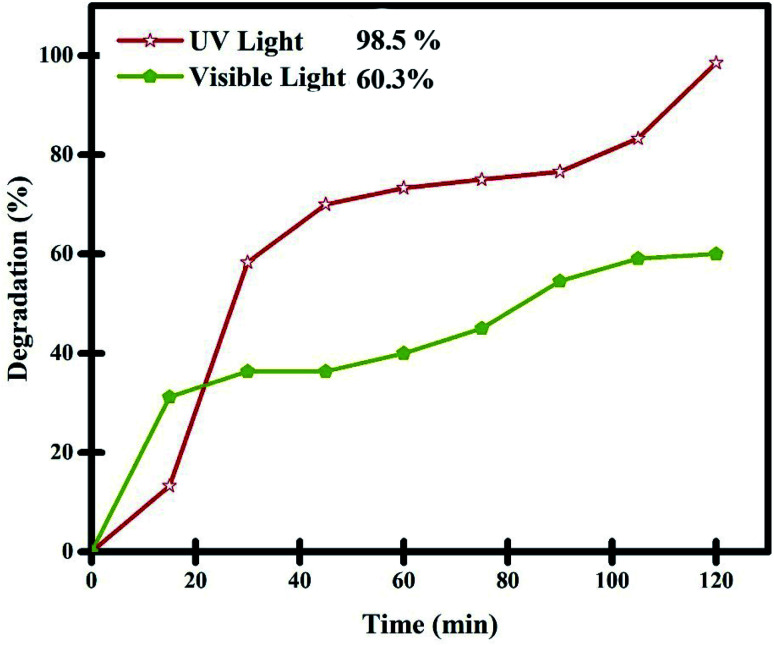
Comparison of the photocatalytic performance of Lu_2_Cu_2_O_5_–Lu_2_O_3_ nanobundles under UV and Vis irradiation for the degradation of thymol blue.

To discover the most appropriate conditions for the highest degradation efficiency of thymol blue contaminant by the nanocomposite sample, we evaluated the effects of various factors, such as the quantity of photocatalytic nanostructure, the concentration of the contaminant compound and the kind of light source (Fig. 10 and 11 and Table 2). Fig. 10a–c reveals the effects of the Lu_2_Cu_2_O_5_–Lu_2_O_3_ catalyst dosage (0.03, 0.05, and 0.07 g) and the concentration of the contaminant compound in the photocatalytic degradation of thymol blue under UV light irradiation. It can be seen that, to purify a 10 ppm thymol blue contaminant solution, the use of 0.05 g of Lu_2_Cu_2_O_5_–Lu_2_O_3_ nanobundles leads to the highest degradation rate of 98.5% (Fig. 10b). To purify thymol blue solution with a concentration higher than 10 ppm (15 ppm), the use of 0.03 g and 0.07 g of the nanocomposite sample brings the same photocatalytic efficiency (96.6%). When increasing the concentration of thymol blue solution to 20 ppm, the use of 0.03 g of nanocomposite sample leads to the highest percentage of degradation (75%). Thus, the above outcomes indicate that the use of 0.05 g of Lu_2_Cu_2_O_5_–Lu_2_O_3_ nanobundles is the best option to purify thymol blue solution with a concentration of 10 ppm and achieve the highest degradation efficiency (Fig. 10) because, in these conditions, the adsorption of contaminant molecules on the surface of Lu_2_Cu_2_O_5_–Lu_2_O_3_ nanobundles is better and the penetration of light into the contaminant solution is more appropriate.^12,40^

The performance of the nanocomposite sample in the purification of aqueous thymol blue contaminant under visible light was also evaluated (Fig. 11). In this experiment, 0.05 g of Lu_2_Cu_2_O_5_–Lu_2_O_3_ nanobundles was employed to purify a solution with a concentration of 10 ppm thymol blue. It was observed that about 59.1% of thymol blue contaminant can be removed by the nanocomposite sample within 120 minutes of visible light irradiation. The performance of the Lu_2_Cu_2_O_5_–Lu_2_O_3_ nanobundles in the removal of thymol blue contaminant under visible light is lower than that under ultraviolet light, which confirms the DRS results.

### 3.6. Photocatalytic mechanism for the removal of thymol blue

**Fig. 12:**
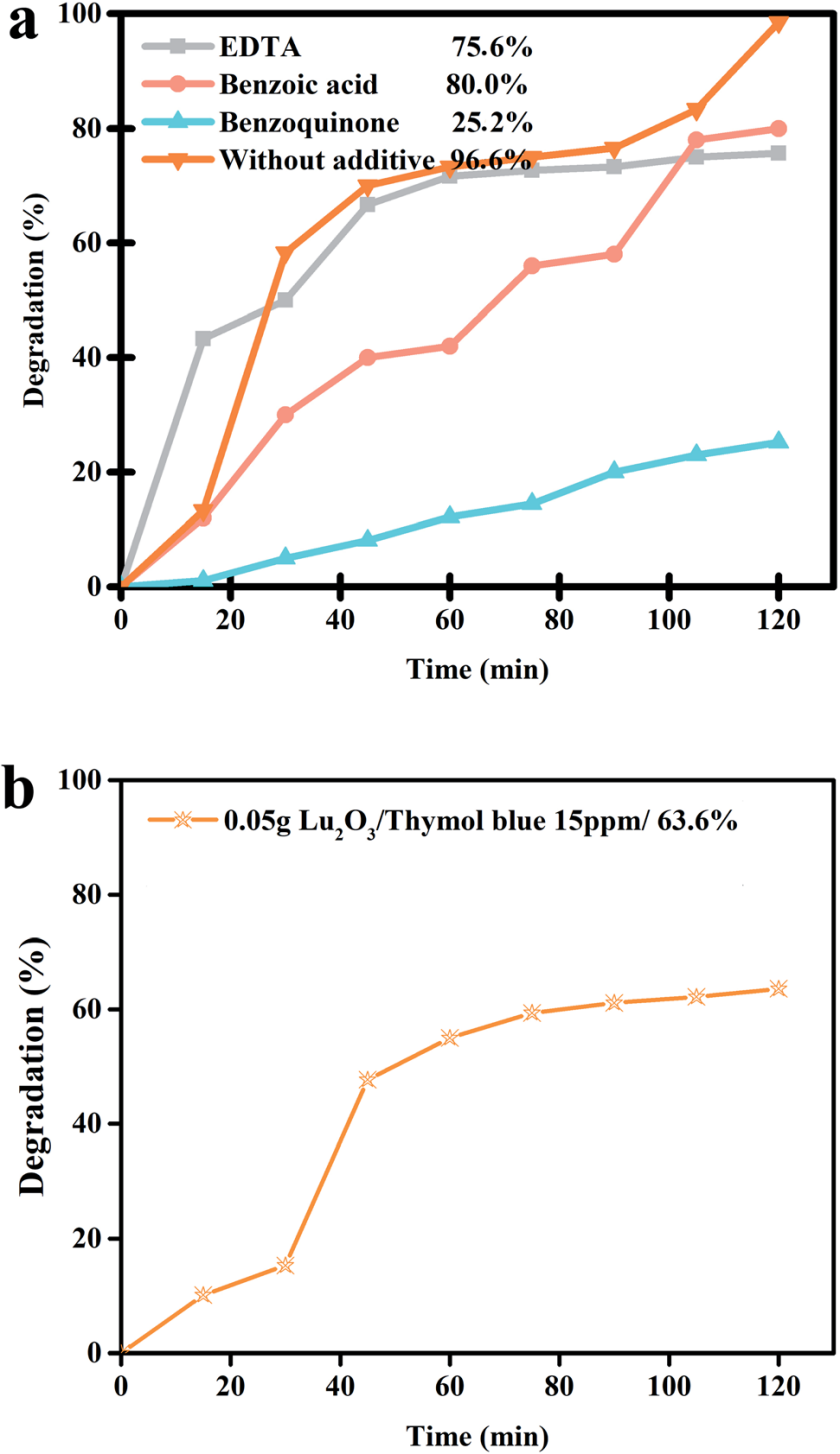
(a) Radical trapping experiment of active species in the photocatalytic degradation of thymol blue over Lu_2_Cu_2_O_5_–Lu_2_O_3_ nanobundles and (b) photocatalytic activity of Lu_2_O_3_ over thymol blue.

Scavenging experiments were undertaken separately to detect the reactive radical species that are significantly responsible for decomposing the thymol blue contaminant (Fig. 12a). It was observed that after the addition of benzoic acid and EDTA, the photocatalytic performance of the nanocomposite sample does not alter significantly compared to the scavenger-free system. This signifies that the hole and the hydroxyl radicals cannot play a meaningful role in the photocatalytic degradation of thymol blue contaminant in the presence of the nanocomposite sample. However, the introduction of benzoquinone can dramatically suppress the photocatalytic efficiency of Lu_2_Cu_2_O_5_–Lu_2_O_3_ nanobundles, indicating that O_2_^−^˙ is the leading active species for thymol blue removal in the presence of the nanocomposite sample. The catalytic reactions for the removal of thymol blue contaminant by Lu_2_Cu_2_O_5_–Lu_2_O_3_ nanobundles through hydroxyl radicals, which can be generated by the leading active species, O_2_^−^ radicals, can be summarized as follows.^41–44^Lu_2_Cu_2_O_5_–Lu_2_O_3_ nanobundles + *hν* → Lu_2_Cu_2_O_5_–Lu_2_O_3_ nanobundles* (e_CB_^−^ + h_VB_^+^)H^+^ + O_2_˙^−^ → HO_2_˙O_2_ + e^−^ → ˙O_2_^−^h^+^ + H_2_O → ˙OH + H^+^˙O_2_^−^ + e^−^ + H^+^ → HOO˙HOO˙ + H_2_O → ˙OH + H_2_O_2_2H^+^ + ˙O_2_^−^ → H_2_O_2_H_2_O_2_ + e^−^ → ˙OH + OH^−^OH^−^ + h^+^ → ˙OHH_2_O + h^+^ → ˙OH + H^+^OH˙ + thymol blue → degradation products (*e.g.*, H_2_O, CO_2_)e_CB_^−^ + thymol blue → reduction productsh_VB_^+^ + thymol blue → oxidation products

Fig. 12b reveals the photocatalytic activity of Lu_2_O_3_ over thymol blue, indicating it degraded this colorant about 63.0%, which is much less than the degradation by Lu_2_Cu_2_O_5_–Lu_2_O_3_ nanocomposites. This result showed that Lu_2_Cu_2_O_5_–Lu_2_O_3_ nanocomposite has better activity in the photodegradation of organic dyes.

### 3.7. Stability and recyclability of Lu_2_Cu_2_O_5_–Lu_2_O_3_

**Fig. 13:**
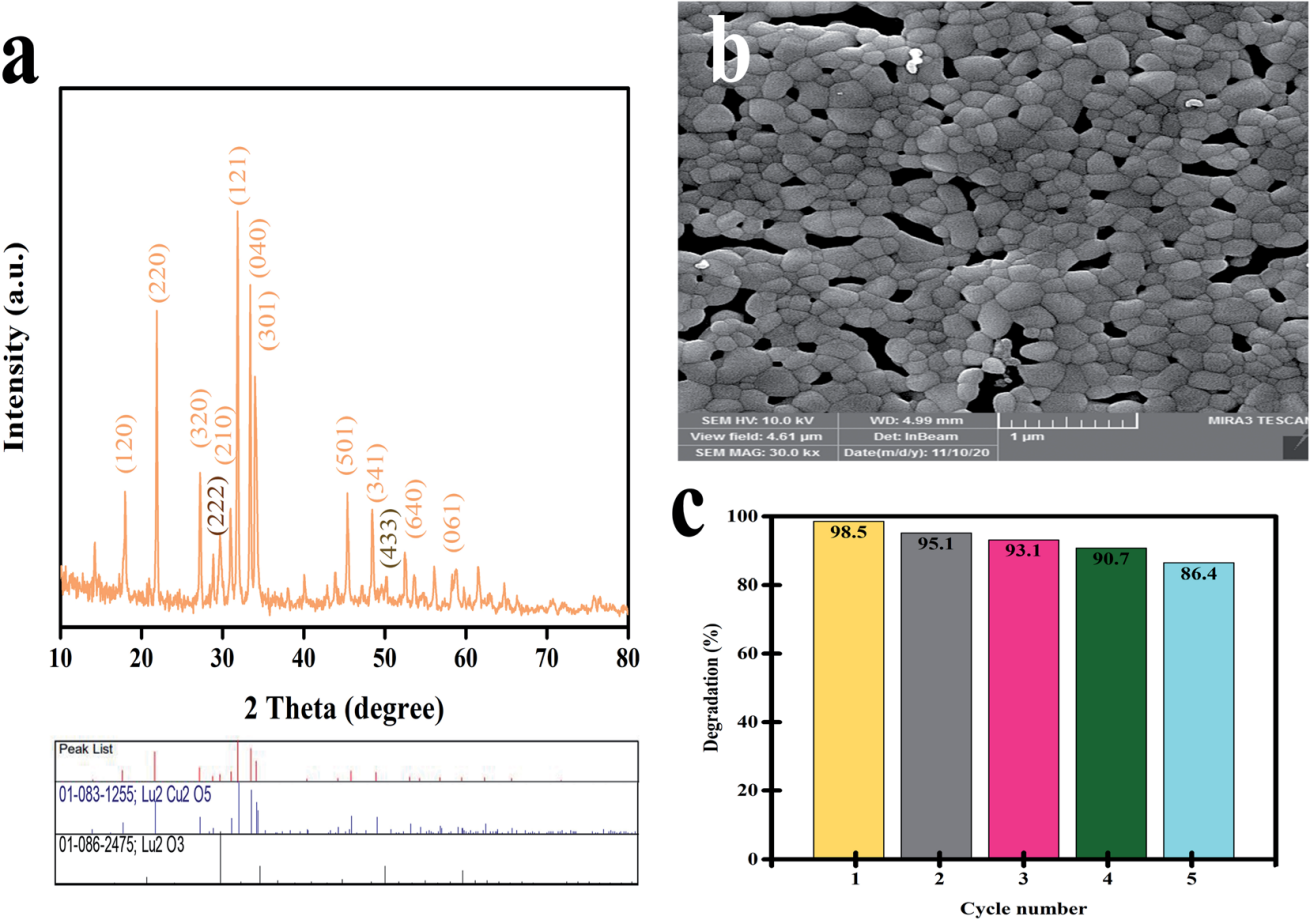
(a) XRD pattern and (b) FESEM image of Lu_2_Cu_2_O_5_–Lu_2_O_3_ after photocatalytic reaction after 5 runs, and (c) cycling runs in the photocatalytic degradation of 10 ppm thymol blue under visible irradiation.

The stability of Lu_2_Cu_2_O_5_–Lu_2_O_3_ was studied by XRD and SEM analysis after photodegradation. Fig. 13a shows the XRD pattern of the as-prepared catalyst after 5 runs. This pattern shows that Lu_2_Cu_2_O_5_ (reference code 83-1255) and Lu_2_O_3_ (reference code 086-2475) were present, indicating the high stability of this composite. On the other hand, the FRSEM image shows that the particles are attached to each other and agglomeration has occurred (Fig. 13b). To check the recyclability of Lu_2_Cu_2_O_5_–Lu_2_O_3_, the catalyst was centrifuged, washed with ethanol and water, dried at 65 °C for 24 h, and reused five times under the same conditions. As shown in Fig. 13c, Lu_2_Cu_2_O_5_–Lu_2_O_3_ is very stable and maintains its high photocatalytic performance across five reaction cycles. Indeed, during the fifth cycle, the reduction in photocatalytic activity is only 12.1%.

## 4. Conclusions

In this work, a new type of nanostructure, Lu_2_Cu_2_O_5_–Lu_2_O_3_ nanocomposite, was successfully designed and fabricated through a quick and eco-friendly approach. Morphological studies demonstrated that the use of 5 mmol of amylum, an appropriate green reactant, was the best option for the efficient synthesis of Lu_2_Cu_2_O_5_–Lu_2_O_3_ nanobundles with the most organized morphology. The features of Lu_2_Cu_2_O_5_-based nanostructures were carefully investigated utilizing multiple characterization methods. With an energy gap of 3.2 eV, the catalytic role of the fabricated nanobundles was evaluated for the removal of various kinds of toxic contaminants. The influences of the quantity of photocatalytic nanostructure, the concentration of the contaminant compound and the type of light source in the catalytic degradation process were evaluated. The highest photocatalytic performance was observed for 0.05 g of Lu_2_Cu_2_O_5_–Lu_2_O_3_ nanobundles, with an efficiency of 98.5% in 2 hours for degradation of the contaminant thymol blue at a concentration of 10 ppm under ultraviolet light irradiation. Experimental results demonstrated that superoxide radicals were largely responsible for the elimination of the toxic pollutant through the photocatalytic pathway. To our knowledge, this is the first investigation of the fabrication of Lu_2_Cu_2_O_5_–Lu_2_O_3_ nanocomposite through a facile and eco-friendly auto-combustion approach and of its photocatalytic efficiency. Exploitation of environmentally friendly and available reactants as well as the implementation of an efficacious fabrication method are the highlights of this research and make Lu_2_Cu_2_O_5_–Lu_2_O_3_ nanocomposite a superior candidate for environmental remediation.

## Conflicts of interest

The authors declare that there are no conflicts of interest regarding the publication of this manuscript.

## Supplementary Material
